# A Novel Approach on Drug Delivery: Investigation of A New Nano-Formulation of Liposomal Doxorubicin and Biological Evaluation of Entrapped Doxorubicin on Various Osteosarcoma Cell Lines

**DOI:** 10.22074/cellj.2017.4502

**Published:** 2017-05-17

**Authors:** Fateme Haghiralsadat, Ghasem Amoabediny, Mohammad Hasan Sheikhha, Tymour Forouzanfar, Marco N. Helder, Behrouz Zandieh-doulabi

**Affiliations:** 1 Department of Life Science Engineering, Faculty of New Sciences and Technologies, University of Tehran, Tehran, Iran; 2 Department of Nano Biotechnology, Research Center for New Technologies in Life Science Engineering, University of Tehran, Tehran, Iran; 3 Department of Biotechnology and Pharmaceutical Engineering, School of Chemical Engineering, College of Engineering, University of Tehran, Tehran, Iran; 4 Department of Oral and Maxillofacial Surgery, VU University Medical Center, MOVE Research Institute Amsterdam, Amsterdam, The Netherlands; 5 Research and Clinical Center for Infertility, Shahid Sadoughi University of Medical Sciences, Yazd, Iran; 6 Biotechnology Research Center, International Campus, Shahid Sadoughi University of Medical Science, Yazd, Iran; 7 Oral Cell Biology and Functional Anatomy, Academic Centre for Dentistry Amsterdam (ACTA), University of Amsterdam and Vrije Universiteit Amsterdam, Amsterdam, The Netherlands

**Keywords:** Chemotherapy, Characterization, Osteosarcoma, Liposomal Doxorubicin

## Abstract

**Objective:**

In this study we prepared a novel formulation of liposomal doxorubicin (L- DOX). The drug dose was optimized by analyses of cellular uptake and cell viability of osteosarcoma (OS) cell lines upon exposure to nanoliposomes that contained varying DOX concentrations. We intended to reduce the cytotoxicity of DOX and improve characteristics of the nanosystems.

**Materials and Methods:**

In this experimental study, we prepared liposomes by the pH gradient hydration method. Various characterization tests that included dynamic light scattering (DLS), cryogenic transmission electron microscopy (Cryo-TEM) imaging, and UV- Vis spectrophotometry were employed to evaluate the quality of the nanocarriers. In addition, the CyQUANT® assay and fluorescence microscope imaging were used on various OS cell lines (MG-63, U2-OS, SaOS-2, SaOS-LM7) and Human primary osteoblasts cells, as novel methods to determine cell viability and *in vitro* transfection efficacy.

**Results:**

We observed an entrapment efficiency of 84% for DOX within the optimized liposomal formulation (L-DOX) that had a liposomal diameter of 96 nm. Less than 37% of DOX released after 48 hours and L-DOX could be stored stably for 14 days. L-DOX increased DOX toxicity by 1.8-4.6 times for the OS cell lines and only 1.3 times for Human primary osteoblasts cells compared to free DOX, which confirmed a higher sensitivity of the OS cell lines versus Human primary osteoblasts cells for L-DOX. We deduced that L- DOX passed more freely through the cell membrane compared to free DOX.

**Conclusion:**

We successfully synthesized a stealth L-DOX that contained natural phospholipid by the pH gradient method, which could encapsulate DOX with 84% efficiency. The resulting nanoparticles were round, with a suitable particle size, and stable for 14 days. These nanoparticles allowed for adequately controlled DOX release, increased cell permeability compared to free DOX, and increased tumor cell death. L-DOX provided a novel, more effective therapy for OS treatment.

## Introduction

Cancer is the cause of a majority of serious health problems and mortality worldwide. Osteosarcoma (OS), the most common primary malignant tumor of the bone, has a worldwide incidence of approximately 1 to 3 cases annually per million, and comprises almost 60% of all bone sarcomas (1,2). Currently, researchers are pursuing new methods to develop a sufficient, effective treatment (3,4). Chemotherapy, ahead of other conventional treatments, has taken the main role in treating cancer (5). Doxorubicin (DOX) is a small molecular, broad-spectrum chemotherapeutic drug widely used to treat various cancers. DOX therapeutic activity is achieved through intercalation into DNA thereby inhibiting topoisomerase-II function (6). Although DOX is used as a general anti-cancer drug, it has shown an excellent therapeutic efficacy on OS (7). However, DOX has a very narrow therapeutic index (8-10). Often the administration of DOX comes with a number of undesirable adverse effects such as cardiotoxicity, myelosuppression, typhlitis, nausea, vomiting, and alopecia. These adverse effects can be decreased by administration of DOX through a designed, specialized delivery system that eliminates drug accumulation at unnecessary sites. Various researchers have studied DOX capsulation methods as a drug delivery strategy to cancer tissues in an attempt to diminish cardiotoxicity, as the most prominent adverse effect (7,11). Over the past decade, nanotechnology has been recognized as one of the most promising tools of cancer management (10,12-14). Enhancing drug solubility, stability, and plasma half-life are a few of its many applications in the pharmaceutical industry, particularly in terms of nanodelivery systems (NDS) (15,16). Such systems have enabled us to change unfavorable physicochemical properties of bioactive molecules, improve delivery of therapeutics across biological barriers and compartments, control release of bioactive agents, and enhance therapeutic efficacy by selective delivery to biological targets (17-20). One of the first and most important nano-based platforms that has been applied in medicine is liposome technology (21,22). These spherical nano-scaled vesicles consist of an aqueous core and a vesicle shell with either single or multiple bilayer membrane structures composed of natural or synthetic lipids (23). The mentioned structure allows encapsulation of hydrophilic agents in their aqueous core and hydrophobic ones within their lamellae (24). In order to improve stability and circulation half-life, liposomes may be coated with suitable polymer coatings such as polyethylene glycol (PEG), thus creating PEGylated liposomes (21). PEG coating also helps to reduce systemic phagocytosis, which results in prolonged systemic circulation, selective agent delivery through leaky tumor endothelium, permeability and retention effect enhancement, as well as reduced toxicity profiles (25). 

In this research, we attempted to enhance DOX importation into several OS cell lines by developing a novel PEGylated DOX- loaded liposome to heighten the intracellular nanosystem uptake. We analyzed this nanosystem in an attempt to improve treatment efficacy and, in particular, reduce toxicity. We also employed this novel method to decrease the toxicity of DOX against normal cells by dose optimization of DOX. 

## Materials and Methods

DOX hydrochloride (DOX-HCl) was obtained from Ebewe Pharma (Austria). Egg phosphatidylcholine (EPC), and derivatized distearyl phosphatidylethanolamine (mPEG2000- DSPE) were obtained from Lipoid GmbH (Ludwigshafen, Germany). Cholesterol (CHOL), fluorescent label (Dil), and phosphate-buffered saline (PBS, pH=7.0) were purchased from Sigma- Aldrich Co. (St. Louis, MO, USA). Dialysis bags (MWCO 12000-14000) were supplied by Jingkehongda Biotechnology Co., Ltd. (Beijing, China). Chloroform, ethanol and other chemicals used in this study were analytical grade. 

### Preparation of liposomes

**Fig.1:**
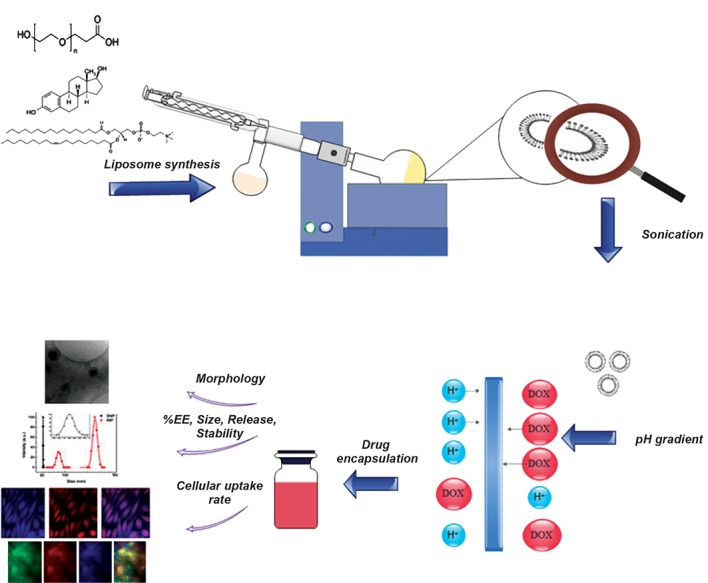
A brief overview of the research work flow: synthesis, doxorubicin (DOX) loading and liposome characterization. EE; Encapsulation efficiency.

We used the pH gradient hydration method to prepare the liposomes. EPC, CHOL, and DSPE- mPEG (67.9:29.1:3 molar ratios) were dissolved in chloroform, and the mixture was warmed to 55˚C. We added Dil to the lipid phase at 0.1 mol% for lipid staining in order to evaluate cellular uptake. The solvent was evaporated under vacuum in a rotary evaporator until a thin-layered film formed. Once prepared, the film was hydrated with 250 mM of ammonium sulfate. The hydrated film was extruded 5 times through 0.2 μm pore size polycarbonate membranes, and 5 times through 0.1 μm pore size polycarbonate membranes using a mini-extruder in order to decrease liposomal particle size. Various doses of DOX (0.2, 0.5, 1, and 1.5 mg/ml) were loaded into the liposomes. Afterwards, free DOX (unloaded) was separated from liposomal DOX (L-DOX) by dialysis bags that had a cut-off of 12 kDa. Figure 1 shows a schematic diagram of the experimental set-up. 

### Determination of size and polydispersity index

The particle size distribution and polydispersity index (PDI) of the liposome particles was determined at 25˚C by dynamic light scattering (DLS) using a ZetaPALS zeta potential and particle size analyzer (Brookhaven Instruments, Holtsville, NY, USA). Each parameter was measured three times, after which we calculated average values and standard deviations. L-DOX morphology was investigated by cryogenic transmission electron microscopy (Cryo-TEM, FEI Tecnai 20, type Sphera, Hillsboro, OR, USA). 

### Determination of doxorubicin-loaded efficiency 

The L-DOX particles were lysed with Triton X-100 to analyze DOX concentrations. The DOX encapsulation efficiency of the L-DOX collected fractions was determined by measuring absorption at 480 nm with a UV-Vis spectrophotometer (model T80+, PG Instruments, UK). In order to determine the encapsulation efficiency, we used the following formula: 

Encapsulation efficiency %= ( Loaded drug in liposome (mg))/( Total drug (mg))×100

### Stability studies 

We evaluated stability of the L-DOX suspension by particle size analysis and drug encapsulation efficiency of the samples after storage at 4˚C for 14 days. 

### *In vitro* release studies of doxorubicin from liposomes

We used dialysis bags (MWCO 12000) to monitor the amount of drug released from the liposomes against PBS (dialysis medium) for 48 hours at 37˚C and pH=7.0. Samples of medium were taken at different times and replaced with the same volume of PBS to evaluate the DOX release rate from the liposomes. Samples were analyzed via UV-Vis spectrophotometry at 480 nm. UV absorption showed a linear response in the range of 0.5-50 µg/ml. For each sample we calculated the total concentration of drug loaded in a liposome formulation and percentage of released drug. 

### Morphology evaluation

The nanoparticle bilayer structure and round shape was examined by Cryo-TEM (FEI Tecnai 20, type Sphera, OR, USA) at 200 kV. 

### Cell lines and cultures

RPMI-1640 cell culture medium was purchased from Gibco, Invitrogen (GmbH, Karlsruhe, Germany). 4´,6-diamidino-2-phenylindole (DAPI) and dimethyl sulfoxide (DMSO) were provided by Thermo Fisher Scientific (Waltham, MA, USA) and Sigma-Aldrich (St. Louis, MO, USA). The MG-63 cell line was supplied by Pasteur Institute (Iran). The SaOS-2 cells were kindly provided by Dr. F. van Valen (Westfalische Wilhelms-Universität, Münster, Germany), and U2-OS by Dr. S. Lens (Dutch Cancer Institute, Amsterdam, the Netherlands). SaOS-LM7 cells were kindly provided by Dr. E.S. Kleinerman (MD Anderson Cancer Center, Houston, TX, USA). Human primary (short-term culture: passage<10) osteoblasts Human 54 were obtained from healthy patients undergoing total knee replacement after they provided informed consent. All cells, with the exception of SaOS-LM7, were cultured in RPMI-1640 medium (Gibco, Invitrogen, GmbH, Germany) supplemented with 10% fetal calf serum (FCS), and penicillin/streptomycin (1 mg/mL, Pen- Strep, Gibco, Invitrogen, Germany) at 37˚C and 5% CO_2_ in a humidified incubator. LM7 was cultured in E-MEM (Lonza) supplemented with 10% FCS, 1 mg/mL Pen-Strep, 1% non- essential amino acids, 1% sodium pyruvate, 2 nM L-glutamine, and 2% MEM vitamin solution (all: Gibco, Invitrogen, Germany) at 37˚C and 5% CO_2_ in a humidified incubator. DOX was diluted in RPMI-1640 to the desired concentrations prior to use. 

### Cell viability studies

Human primary osteoblasts cells and the OS cell lines (MG-63, U2-OS, SaOS-2, SaOS- LM7) were seeded on 96-well plates for one day to allow for cell attachment. We replaced the media with media that contained a dilution series of: i. Blank liposomes, ii. DOX, or iii. L-DOX. DOX concentrations varied from 0.1 to 10 mg/mL in (ii) and (iii). Cells were further incubated for 72 hours. Then, the cells were washed with PBS. After removal of PBS, we added 300 μl/well of CyQUANT® lysis buffer supplied with the CyQUANT® kit, and samples were subsequently stored at -20˚C until further analysis. We performed 3 freeze and thaw cycles for the samples. Then, total DNA of duplicate samples was measured with the CyQUANT® cell proliferation assay kit according to the specifications provided by the manufacturer (Molecular Probes Inc., Life Technologies). After exposure, fluorescence measurements were determined using a SynergyTMHT multi-mode microplate reader (Biotek Instruments, Inc.). 

### *In vitro* transfection experiment

We allowed 5×10^5^ SaOS-2 and MG-63 cells to grow in six-well plates in a monolayer for 24 hours. Cells were washed with RPMI-1640 medium and incubated with L-DOX (10 μg/ml) for transfection for 3 hours at 37˚C. The cells were rinsed three times with PBS and fixed with a 4% paraformaldehyde solution (Thermo Scientific, USA). For nuclear counterstaining, we used DAPI (0.125 µg/mL) for 15 minutes. Cellular uptake of DOX was evaluated with a fluorescence microscope (BX61, Olympus, Japan). 

## Results

### Effects of doxorubicin doses

High encapsulation efficiency of a low dose therapeutic agent is an important parameter for enhancement efficiency of large-scale production of the nanodrug, and in terms of reducing toxicity against normal cells. Table 1 shows the effects of various DOX doses as a model of a hydrophilic drug. The encapsulation efficiency increased with decreasing DOX doses. Decreasing the drug dose slightly increased the zeta potential. The drug dose did not affect agglomeration of the vesicle. The vesicle size decreased with decreasing drug doses. We selected the 0.5 mg/ml dose of DOX for further analysis. 

### Characterization of liposome formulation with the optimized doxorubicin dose

**Fig.2:**
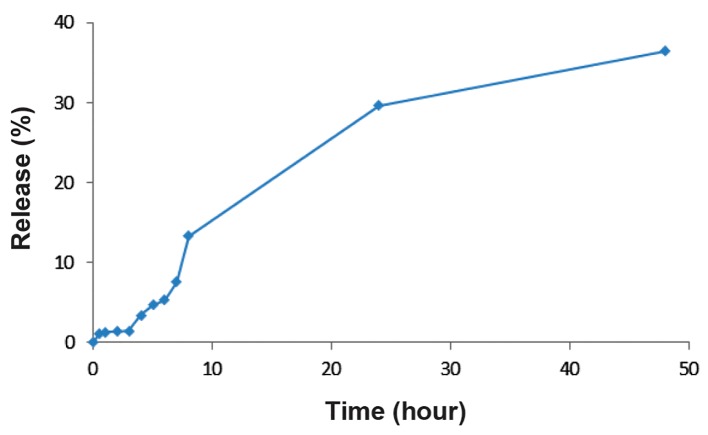
release of liposomal doxorubicin (L-DOX) for 48 hours after drug encapsulation.

The obtained results showed an average 96 nm nanoparticle diameter and -30 mV zeta potential. L-DOX stability tests showed only a slight change (~7% increase) in diameter size (103 nm) with no morphological changes for L-DOX, which confirmed the stability of the presented nanoformulation. There was an 84.32% encapsulation efficiency of DOX into the liposomal vesicle, which slightly reduced (less than 2%) to 83.09% after storage. In the *in vitro* release studies, we observed 37% release of DOX after 48 hours. The drug release profile increased with a gentle slope (Fig.2). 

**Fig.3:**
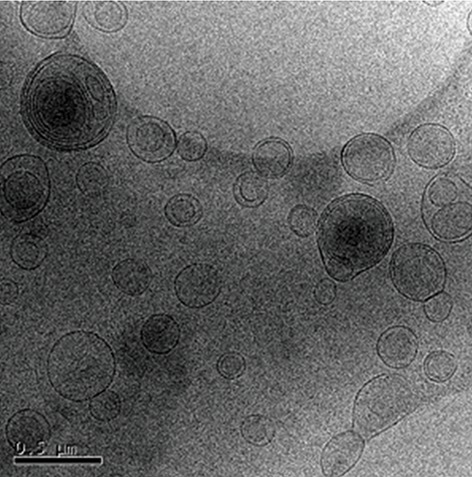
Morphological evaluation of liposomal doxorubicin (L- DOX) by cryogenic transmission electron microscopy (Cryo-TEM) (micrograph bar: 0.5 μm), DOX encapsulated in liposome vesicle with 84% efficiency.

Figure 3 shows the Cryo-TEM micrograph of the liposome vesicles. The biliary structure (hydrophobic and hydrophilic spaces) of liposomes was clear. DOX was entrapped inside the bilayer membrane, restricted to the hydrophilic part of the liposomal vesicle. Cryo- TEM analysis confirmed the low diameter size and round vesicle shape. 

**Table 1:** Effect of various doxorubicin (DOX) doses of the prepared liposome formulation


Formula	Drug dose (mg/ml)	EE (%)	Size (nm)	PDI	Zeta potential (mv)

F1	1.5	59.52 ± 2.86	124 ± 6	0.23 ± 0.05	-21 ± 1.7
F2	1	65.34 ± 7.31	110 ± 5	0.22 ± 0.06	-27 ± 4.3
F3	0.5	84.32 ± 4.87	96 ± 4	0.21 ± 0.04	-30 ± 2.8
F4	0.2	72.56 ± 3.43	91 ± 2	0.2 ± 0.06	-33 ± 1.1


### Cell viability study

**Fig.4:**
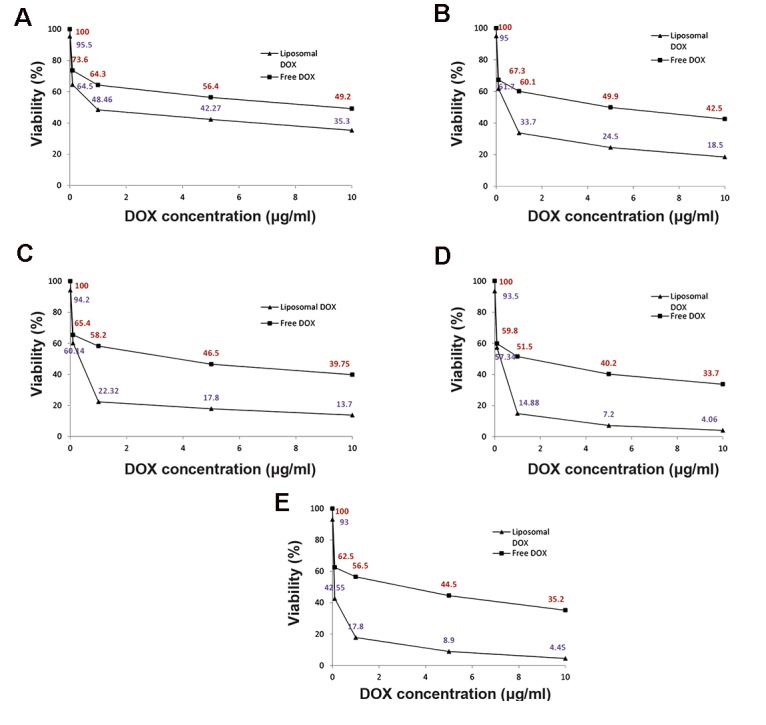
Cell viability assay of various cell lines after 72 hours of treatment with various concentrations of entrapped and free DOX. A. Human primary osteoblasts, B. MG-63 cell line, C. U2-OS cell line, D. SaOS-2 cell line, and E. SaOS-LM7 cell line.

**Fig.5:**
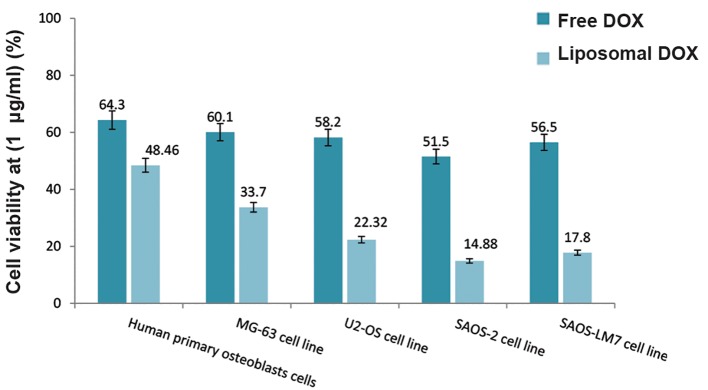
Comparsion of cell viability of various cell lines after 72 hours of treatment with 1 μg/ml of entrapped and free doxorubicin (DOX).

We analyzed the viability of free DOX and L-DOX after 72 hours by the CyQUANT® assay on various cell lines (Fig.4). Empty liposomes caused no toxicity and did not affect the viability of any cell type. As expected, L-DOX induced toxicity on all cell lines at all DOX concentrations. The proposed delivery system for DOX by liposomal formulation increased its toxicity by approximately 1.8 (MG-63), 2.3 (U2-OS), 4.6 (SaOS-2), and 4.4 (SaOS-LM7) times compared to free DOX for these OS cell lines. Toxicity increased 1.3 times for Human primary osteoblasts cells. The half maximal inhibitory concentration (IC_50_) of DOX on all cell lines was 5-10 μg/ml (free DOX) and 0.1-1 μg/ml (L-DOX). Figure 5 provides a comparison of cell viability of DOX (free and liposomal) on various cell lines. The cell viability of free and L-DOX decreased in the following order for the 1 µg/ ml concentration of DOX: SaOS-2 cells>SaOS- LM7 cells>U2-OS cells>MG-63 cells>Human primary osteoblasts cells. We observed a similar trend for the other DOX concentrations. As expected, DOX had fewer side effects on Human primary osteoblasts cells. 

### *In vitro* transfection experiment

**Fig.6:**
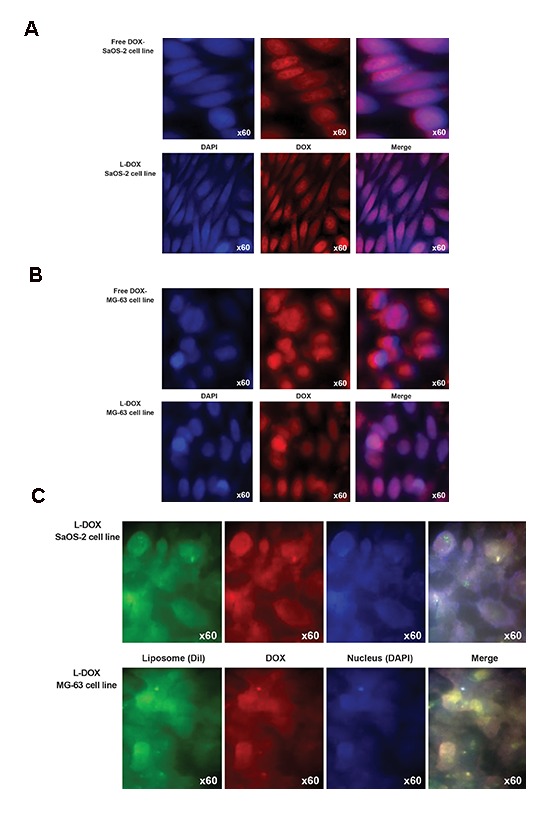
Fluorescence microscope imaging of SaOS-2 and MG-63 cells incubated with doxorubicin (DOX) and liposomal DOX (L-DOX). Cells were treated with DAPI for nucleus staining and Dil dye was used for phospholipid staining. Red: DOX ﬂuorescence, Blue: Fuorescence of the nucleus, Green: Fuorescence of the liposome, Purple: Overlapping ﬂuorescence. A. Comparsion of cellular uptake of DOX and L-DOX for the SaOS-2 cell line, B. Comparsion of cellular uptake of DOX and L-DOX for the MG-63 cell line, and C. Comparsion of cellular uptake of L-DOX for the SaOS-2 and MG-63 cell lines.

Figure 6 shows the *in vitro* transfection image of free DOX and L-DOX on SaOS-2 and MG-63 cell lines monitored by fluorescence microscope. Qualitatively, cells (for both cell lines) treated with L-DOX had greater purple intensity compared to those treated with free DOX. This was particularly observed for the MG-63 cell line. According to Figure 6A and B, greater numbers of cells contained L-DOX. L-DOX transfection showed more DOX distribution in the nuclear region compared to the cytoplasm. Figure 6C confirms the successful cellular uptake of L-DOX for both OS cell lines. Liposome vesicles stained with Dil was accumulated around the nucleus. According to the literature, DOX acts as an apoptosis inducer by down-regulation of topoisomerase II (6). This relies on the proper and timely release of DOX to prevent DNA replication. Figure 6C shows that L-DOX vesicles have successfully passed through the cytoplasm and nuclear membrane. Most importantly, the liposomal membrane degraded only into the nucleus, and not in the cytoplasm or before uptake into the cell. 

## Discussion

DOX is extensively used in cancer therapy and its function has been investigated in cancer biology. The results have presented increasing evidence to support an effective role in cancer treatment. However, the major adverse effects of DOX against normal cells raise serious concerns about its therapeutic safety. The results of the present study demonstrated that a relatively controlled release of DOX through encapsulation in liposomes could reduce L-DOX contact with normal cells. Characteristics of the liposomes included: small size diameter vesicles, high encapsulation efficiency, lack of agglomeration, and stability over a period of 14 days at 4˚C. This has resulted in decreased numbers of adverse effects. Agglomeration is intrinsically inhibited due to the same-sign charge (negative) and mutual repelling force between nanoparticles in the suspension system. 

Establishment of a pH gradient is an effective way to increase encapsulation of any hydrophilic drug into lipid-based vesicles. In this method a small part of DOX is protonated and trapped in the acidic layer of the liposome. The uncharged free base passes through the membrane, establishing a DOX concentration gradient between interior and exterior liposome layers. The proton part therefore acts as a driving force for better diffusion of DOX molecules into the liposomes. Fritze et al. (26) employed the pH-gradient method to improve DOX entrapment efficiency, with a liposomal composition that included a 70:30 EPC and CHOL molar ratio. They examined the release rate of drug at different pH values. Their findings showed that at physiological pH, DOX was retained in L-DOX. Lower pH values increased the DOX release rate. DSPE-mPEG2000 addition to liposomal formulations increased size and *in vivo* stability. Garbuzenko et al. (27) elucidated the effects of PEG–DSPE and CHOL on liposome size. They synthesized liposomes using EPC. Their results showed that 2-7 mol% of DSPE-mPEG increased the diameter size. They also investigated the effect of PEG-DSPE on liposome stability. Their results showed that higher concentrations offered higher stability.

There are several other researches that have attempted to determine a new formulation in order to attain effective encapsulation for DOX. Fang et al. (28) synthesized a magnetic liposomal DOX that contained DSPE-mPEG, EPC, and CHOL by the application of ammonium sulfate gradients through an ethanol injection preparation method. The nanoparticles ranged in size between 50-70 nm and had an encapsulation efficiency of 57.53%. We have chosen a new approach to study the effect of DOX dosing in order to minimize the side effects of DOX against normal cells. Decreasing the amount of drug during preparation of the nanoparticles is advantageous because it becomes more economical to produce while maintaining or even enhancing the appropriate characteristics.

We observed interesting results in the investigations of drug dose effect. These results were likely due to the aqueous capacity of the liposomal vesicle being limited to specific drug amounts, only to be increased by increasing the size of the vesicles. In order to generate low drug dose (0.2 mg/ml) particles, increasing the drug dose to 0.5 mg/ml would generate a strong drug concentration gradient, which supported drug entrapment. Surprisingly, in the case of high drug dosages (1 or 1.5 mg/ml), we observed reduced encapsulation efficiency. We have postulated two possibilities for the decrease in encapsulation efficiency when the liposome membrane is faced with an over-capacity of drug. The drug concentration gradient reverses direction and an anti-encapsulation effect occurs. The amount of drug loaded is the same value for all doses but the denominator (equation 1) has increased. Thus, the encapsulation efficiency deceased. Increased zeta potentials by increased drug dosages might be explained by approaching to the slipping plane.

The liposome formulation synthesized by Egg phosphatidylcholine, CHOL and DSPE-mPEG as the main components, appeared to be nontoxic in the cytotoxicity assays. However when DOX was entrapped, the L-DOX nanoparticles accomplished a more effective tumor cell kill compared to the free form for all OS cell lines. Compared to the OS cells, Human primary osteoblasts cells experienced less toxic effects, as hoped. Apart from differential sensitivity between Human primary osteoblasts cells and OS cells, the controlled release of DOX and targeting towards cells were considered important features for L-DOX formulations that minimized DOX adverse effects. Targeting L-DOX particles appeared possible by the use of natural phospholipid (EPC) due to its high similarity with those present in the cell membrane. Thus, the development of improved L-DOX synthesized by incorporation of egg phospholipid for the treatment of OS appeared useful.

We selected the SaOS-2 and MG-63 cell lines for the cellular uptake assay as models of sensitive, resistant OS cells. According to the cell viability assay, the SaOS-2 cell line showed the most sensitivity for DOX compared to the other OS cell lines, with the MG-63 cell line as the most resistant. Fluoroscopic imaging of particle uptake confirmed this result. There was more intense, widespread purple signal observed in SaOS-2 cells versus MG-63 cells. Interestingly, the morphology of both the SaOS-2 and MG-63 cells changed upon DOX treatment.

There was a slow rate of DOX release from the liposome vesicles (less than 2% within 3 hours). Depicts cells treated with L-DOX within 3 hours for the cellular uptake assay. In comparison with drug release profile results, most of the red ﬂuorescence detected by fluorescence microscopy might be related to L-DOX, but not to DOX already released from liposomes. The negative value for the zeta potential showed that an electrostatic force did not have any influence on the enhancement of cellular uptake due to the negatively charged cytoplasm.

## Conclusion

We successfully synthesized a stealth L-DOX that contained natural phospholipid (EPC) by the pH gradient method, which could encapsulate DOX (0.5 mg/ml) with 84% efficiency. The resulting nanoparticles had a round shape, suitable particle size of 96 nm, 14-day stability, adequately controlled DOX release (37% DOX after 48 hours), increased cell permeability compared to free DOX, and increased tumor cell kill. Hence, they could provide a novel, more effective therapeutic for OS treatment.
